# Postoperative outcomes of elderly patients undergoing partial nephrectomy

**DOI:** 10.1038/s41598-021-96676-y

**Published:** 2021-08-25

**Authors:** Alexandre Ingels, Sophie Duc, Karim Bensalah, Pierre Bigot, Philippe Paparel, Jean-Baptiste Beauval, Laurent Salomon, Alexandre De La Taille, Hervé Lang, François-Xavier Nouhaud, José Batista Da Costa, Charles Dariane, Hervé Baumert, Morgan Roupret, Thibaut Waeckel, Cédric Lebacle, Jean-Alexandre Long, François Henon, Jean-Jacques Patard, Nicolas Doumerc, Arnaud Mejean, Marie-Neige Videau, Jean-Christophe Bernhard

**Affiliations:** 1 Department of Urology, University Hospital Henri Mondor. APHP. UPEC. AP-HP, 51, Avenue du Maréchal de Lattre de Tassigny, 95010 Créteil Cédex, France; 2 Department of Geriatrics, Pôle de Gérontologie Clinique Xavier Arnozan, CHU Bordeaux, Bordeaux, France; 3 Department of Urology, University of Rennes, Rennes, France; 4 Department of Urology, Angers University Hospital, Angers, France; 5 Department of Urology, Lyon Sud-Pierre Bénite Teaching Hospital, Lyon, France; 6 Department of Urology, Saint Jean Languedoc/La Croix du Sud Hospital, Toulouse, France; 7 Department of Urology, Hôpital Mont-de-Marsan, Mont-de-Marsan, France; 8 Department of Urology, University Hospital of Strasbourg, Strasbourg, France; 9 Department of Urology, Rouen University Hospital, Rouen, France; 10 Department of Urology, Assistance Publique Hôpitaux de Paris, Hôpital Européen Georges Pompidou, Paris, France; 11 Department of Urology, Paris Saint-Joseph Hospital, Paris, France; 12 Department of Urology, Sorbonne University, APHP, Pitié-Salpêtrière Hôpital, Paris, France; 13 Department of Urology, Caen University Hospital, Caen, France; 14 Department of Urology, Hôpitaux Universitaires Paris-Sud, Le Kremlin-Bicêtre, France; 15 Department of Urology, Grenoble University Hospital, Grenoble, France; 16 Department of Urology, Lille University, Lille, France; 17 Department of Urology, Kidney Transplantation and Andrology, Toulouse Rangueil University Hospital, Toulouse, France; 18 Department of Urology, Hôpital Pellegrin, Bordeaux University Hospital, Bordeaux, France

**Keywords:** Cancer, Renal cancer

## Abstract

To describe clinical outcomes of patients aged 75 years and above after partial nephrectomy (PN), and to assess independent factors of postoperative complications. We retrospectively reviewed information from our multi-institutional database. Every patient over 75 years old who underwent a PN between 2003 and 2016 was included. Peri-operative and follow up data were collected. Multivariate logistic regression was performed to determine independent predictive factors of postoperative complications. We reviewed 191 procedures including 69 (40%) open-surgery, and 122 (60%) laparoscopic procedures, of which 105 were robot-assisted. Median follow-up was 25 months. The mean age was 78 [75–88]. The American Society of Anesthesiologist’s score was 1, 2, 3 and 4 in 10.5%, 60%, 29% and 0.5% of patients respectively. The mean tumor size was 4.6 cm. Indication of PN was elective in 122 (65%) patients and imperative in 52 patients (28%). The median length of surgery was 150(± 60) minutes, and the median estimated blood loss 200 ml. The mean glomerular filtration rate was 71.5 ml/minute preoperatively, and 62 ml/min three months after surgery. The severe complications (Clavien III-V) rate was 6.2%. On multivariate analysis, the robotic-assisted procedure was an independent protective factor of medical postoperative complications (Odds Ration (OR) = 0.31 [0.12–0.80], p = 0.01). It was adjusted for age and RENAL score, robotic-assisted surgery (OR = 0.22 [0.06–0.79], p = 0.02), and tumor size (OR = 1.13 [1.02–1.26], p = 0.01), but the patients age did not forecast surgical complications. Partial nephrectomy can be performed safely in elderly patients with an acceptable morbidity, and should be considered as a viable treatment option. Robotic assistance is an independent protective factor of postoperative complications.

## Introduction

Renal cell carcinoma (RCC) represents 3% of all cancer, with an increasing incidence in western countries^1^. The median age at diagnosis is 64 years old and over 20% of new cases are discovered after the age of 75^2^. The estimated incidence increases particularly in the older population. Indeed, the age-standardized rates per 10,000 is 0.5 below the age of 40 years old and 35.0 over 75 years old^3^. Partial nephrectomy (PN) is the gold standard for cT1 renal masses management. In comparison with radical nephrectomy, it offers equivalent oncological outcomes and improvement of renal function. It reduces the risk of chronic kidney disease (CKD). Hence, it is recommended whenever technically feasible^4^. Active surveillance can be considered as an alternative for small renal masses (< 2 cm), particularly for frail patients^5^. While older patients are more likely to harbor more aggressive RCC with higher cancer-specific mortality^5^, PN is under-represented in this population, even among patients presenting alteration of the renal function. While PN appears under-utilized among older patients population, the general population follows the opposite trend with an increasing application^6^. This age-specific management can be explained by the competing risks of RCC and surgery, in a population with presumably more comorbidities. There is currently little data in the literature about the specific risks of PN in elderly patients, in terms of morbidity.

This study aims to depict PN outcomes, in terms of complications, and renal function in an over 75 yr(s) old population. It also seeks to identify independent factors of complications, in order to improve the selection of elderly patients eligible for partial surgery. The main outcome of interest was the safety of the procedure, analyzed through the surgical and medical complications occurring up to 3 months after the procedure.

## Material and methods

### Patients

This is a retrospective analysis of a multi-institutional, prospectively-maintained dataset of patients treated with PN for renal tumors between 2003 and 2016 at 15 French centers that are involved in the French network of research on kidney cancer UroCCR (NCT03293563). Patients > 75 yr(s) old undergoing PN for a localized renal tumor were included. The technical approach for the PN was at the surgeon’s discretion. The study was approved by the Institutional Review Board CPP Sud-Ouest et Outre mer III (DC 2012/108). All methods were performed in accordance to the relevant guidelines and regulations. All patients received oral and written information about the objectives and methodology of the UroCCR project, and informed consent was obtained.

### Data measurements

Based on UroCCR database, we evaluated the patients peri-operative data and the complications up to 3 months after surgery. General demographics information were analyzed along with the main comorbidities.

Predictive operative risk was evaluated with the American Society of Anesthesiologists (ASA) and Eastern Cooperative Oncology Group (ECOG) scores.

The specific tumor characteristics analyzed were the TNM stage and the RENAL and PADUA nephrometry scores.

The technical characteristics of the surgery analyzed were surgical approach, indication for PN (elective or imperative), operative and warm ischemia times, estimated blood loss (EBL) and transfusion.

Regarding the safety, we analyszed the length of stay (LoS), decrease in renal function (estimated glomerular filtration rate (eGFR) calculated with the Modification of Diet in Renal Disease formula (MDRD)and based on the creatinine level measured in µmol/L), intra and post-operative complications reported with the modified Clavien-Dindo classification^7^.

The pathological findings with the RCC histological subtype, pTNM stage, Fuhrmann grade, surgical margins, peri-renal or sinusal fat invasion, renal vein thrombus, microvascular invasion, sarcomatoid component were also analyzed.

The trifecta achievement was assessed according to the Khalifeh criteria: combination of negative surgical margin, no perioperative complications, and ischemia time < 25 min^8^.

### Statistical analysis

Odds ratio (OR) and 95% confidence intervals (CI) were obtained using logistic regression models.

Univariate models were fitted for all clinical and demographic variables. Multivariate logistic regression was performed to determine independent predictive factors of postoperative complications (medical and surgical). A backward stepwise manual method was used for the reduced final multivariate model to predict postoperative complications, adjusted for variables significant at the 0.20 level in univariate analysis and for age, regardless of the significance level in univariate analysis. All analyses were performed in SAS, 9.4.

The significance level alpha was 0.05.

## Results

A total of 191 procedures were reviewed, including 69 (40%) open-surgery and 122 (60%) laparoscopic procedures, of which 105 were robot-assisted. The mean age was 78 [75–88], 60% of patients (n = 117) were men. Median follow-up was 25 months [10.0–31.2]. The ASA score was 1, 2, 3 and 4 in 10.5%, 60%, 29% and 0.5% of patients respectively. One hundred and forty-four patients (75.4%) were asymptomatic at diagnosis. The mean tumor size was 4.6 cm. The indication of PN was elective in 122 (65%) patients and imperative in 52 patients (28%). Median eGFR was 72.7 mL/min/m2 and 51 (26.7%) patients had a pre-operative eGFR < 60 mL/min/m2. The mean length of surgery was 161 (± 60) minutes. The median estimated blood loss was 200 ml and the postoperative transfusion rate was 14.7%. Trifecta was achieved in 45.0% of the procedures. Clinical and surgical data are summarized in Table 1, histological data in Table 2.

**Table 1:** Clinical and surgical data.

Clinical and surgical data	n (%)
Patients, n (%)	191
**Age, years**	
Mean (SD)	78.4 (2.9)
Median [range]	78 [75–88]
Gender male, n (%)	117 (61.3)
Median eGFR before surgery	72.7 mL/min/m^2^
Symptoms at diagnosis, n (%)	47 (24.6)
**ASA scare, n (%)**	
1	19 (10.4)
2	110 (60.1)
3	53 (29.0)
4	1 (0.5)
**ECOG, n (%)**	
0	101 (58.7)
≥ 1	71 (41.3)
Tumor size, cm, mean (SD)	4.7 (3.4)
**Tumor stage**	
1a	87 (45.6)
1b	79 (41.4)
2	13 (6.8)
3a	3 (1.6)
3b	1 (0.5)
Unknown	6 (3.1)
**PADUAL scor**e	
6–8	63 (39.1)
9–11	77 (47.8)
12–14	21 (13.1)
**RENAL score**	
4–6	44 (27.7)
7–9	91 (57.2)
10-Dec	24 (15.1)
**Approach, n (%)**	
Open	69 (40)
Pure laparocopy	17 (8.4)
Robot assisted	105 (51.6)
**Indication of PN, n (%)**	
Elective	122 (65.2)
Imperative	52 (27.8)
Relative	13 (7.0)
Median time of surgery minutes [range]	150 [45–420]
Median estimated blood loss [range]	200 [0–2000]
Median length of stay, days [range]	6 [0–170]

**Table 2:** Histological data of tumors.

Histological data	n (%)	Missing data, n (%)
Clear Cell Renal Cell Carcinoma (RCC)	114 (60)	–
Chromophobe RCC	22 (11.6)	–
Tubulo-papillary RCC	23 (12.1)	–
Oncocytoma	15 (7.9)	–
Simple cyst	7 (3.7)	–
Oncocytic papillary RCC	2 (1.0)	–
Surgical margins	30 (15.9)	–
Invasion of peri-renal or sinusal fat	39 (20.4)	–
Thrombus	9 (4.71)	103 (53.9)
Microvascular invasion	13 (9.6)	–
Sarcomatoid component	5 (2.6)	68 (35.6)
Furhrman grade		45 (23.6)
1	9 (6.1)	
2	73 (49.7)	
3	55 (37.4)	
4	10 (6.80)	

The intra-operative complication rates were 9%, of which most were hemorrhages requiring transfusions. The medical and surgical postoperative complication rates were respectively 19% and 10.5%. Details of the complications are reported in Table 3. According to the modified Clavien-Dindo classification, the severe complications (III-V) rate was 6.2%. On multivariate logistic regression, robotic-assisted procedure was an independent protective factor of both medical (adjusted for age, RENAL score, bilateral location and tumor size) and surgical (adjusted for age and creatinine level in µmol/L) postoperative complications (OR = 0.22 [0.06–0.79], p = 0.02 and OR = 0.31 [0.12–0.80], p = 0.01, respectively). The higher pre-operative creatinine level was associated with increased odds of surgical complication (OR = 1.01 [1.00–1.02], p = 0.04), while the bilateral tumor location (OR = 4.91 [1.04–23.15], p = 0.04) and tumor size (OR = 1.14 [1.02–1.27], p = 0.01) were associated with increased odds of medical complications (Table 4).

**Table 3:** Details of complications.

Intra-operative complications n (%)	17 (9)
Hemorrhage	7 (3.7)
Digestive and vascular wound	3 (1.6)
Pleural breach	3 (1.6)
Conversion	3 (1.6)
Intraoperative transfusion	15 (7.8)
Medical postoperative complications n(%)	36 (19)
Acute renal failure	3 (1.6)
Pneumonia	5 (2.6)
Delirium	3 (1.6)
Ileus	2 (1.0)
Urinary tract infection	4 (2.1)
Sepsis	2 (1.0)
Acute retention of urine	4 (2.1)
Thrombophlebitis	2 (1.0)
Others	11 (5.8)
Surgical postoperative complications n(%)	20 (10.5)
Urinary fistula	2 (1.0)
Perirenal hematoma	7 (3.7)
Wound infection	1 (0.5)
Wall hematoma	2 (1.0)
Arteriovenous fistula	3 (1.6)
False aneurysm	4 (2.1)
Surgical revision	9 (4.7)
Postoperative Transfusion	28 (14.7)
Death	4 (2.1)
Trifecta achievement	45.00%

**Table 4:** Multivariate Logistic Regression of predictive factors of complications.

Predictive factors of surgical postoperative complications	OR (95% CI)	p-value
Age (1 year increment)	1.04 (0.90–1.12)	0.54
Creatinine (1 µmol increment)	1.01 (1.00–1.02)	0.04
Robot-Assisted	0.31 (0.12–0.80)	0.01

Predictive factors of surgical postoperative complications	OR (95% CI)	p-value
Age (1 year increment)	1.04 (0.90–1.12)	0.54
Creatinine (1 µmol increment)	1.01 (1.00–1.02)	0.04
Robot-Assisted	0.31 (0.12–0.80)	0.01
Predictive factors of medical postoperative complications	OR (95% CI)	*p*-value
Age (1 year increment)	1.00 (0.81–1.23)	0.99
Renal Score (1 point increment)	1.02 (0.70–1.49)	0.90
Bilateral Tumor	4.91 (1.04–23.15)	0.04
Robot-Assisted	0.22 (0.06–0.79)	0.02
Tumor Size (1 cm increment)	1.14 (1.02–1.27)	0.01

Among this > 75 yr(s) old population, age was not significantly associated with postoperative complications. The median length of stay at hospital was 6 days. Sixteen patients died, with only 3 deaths related to RCC. Four patients died in the early post-operative period, 2 presented a pulmonary embolism, 2 had a respiratory distress, or complicating decompensation of a chronic obstructive pulmonary disease for the former and asthma for the latter.

Regarding the renal function, the mean pre, per and post-operative (3 months) eGFR were 71.5 ml/min, 64.8 ml/min and 61.8 ml/min. The mean eGFR decrease was 7.1 ml/min (standard deviation 15.8) at 3 months.

## Discussion

In this large retrospective series, we report the feasibility and safety of partial nephrectomy in elderly patients. Nephron-sparing surgery appears to be a valuable option, with a low morbidity rate and good quality outcomes, thus allowing both preservation of renal function and control of the cancer. The results of this study were obtained in spite of the overall challenging presentations of this cohort, with high rates of complex tumors and imperative indications. This procedure should be considered regardless of the patients age—if the individual RCC specific risk is high and competing comorbidities allow a curative strategy. The robot-assisted laparoscopic approach was associated with a low morbidity rate, with similar rates of surgical margins and warm ischemia time.

While partial nephrectomy is the standard of care, and recommended as the first treatment option for T1 tumors with a strong level of evidence^4^, it seems to remain underutilized in elderly patients^9^. Using the Surveillance, Epidemiology, and End Results database, Tan et al. demonstrated that the use of PN vs RN was significantly higher among younger patients. In comparison to the 65–69 yr(s) old age group, the PN odds ratio among the 70–74, 75–79, 80–84 and ≥ 85 yr(s) old patients were 0.82, 0.80, 0.55 and 0.30 respectively^10^. Leppert et al. reported the evolution of PN and RN relative use (2002–2014) from the Veterans Health administration database. While they found an overall shift from 17 to 38% in favor of PN during this period, older patients, and patients with worse baseline kidney function showed the least increase^11^. The large development of this procedure in clinical practices was more limited among older patients, who were likely to remain treated with RN^6,9^. The older patients were nevertheless more likely to present altered renal function at the time of diagnosis. In our study, 26.7% of the patients presented at least a moderate CKD before surgery, and PN was imperative in 27.8% of cases. Therefore, the rationale for PN seems more relevant among elderly patients, where preservation of kidney function is paramount to prevent end-stage renal disease and potential cardiovascular disorders^4^.

The paradoxical underutilization of PN might stem from the surgical risk of a potentially frail population, with more pre-existing comorbidities. Higher rates of peri-operative complications have been reported with PN, in comparison to RN^12,13^. However, in our study, the complication rate was acceptable with a 6.2% of Clavien ≥ III complication rate. This rate is comparable to previous series^8,14^. These results were found despite a high rate of complex tumors with a median tumor size of 4.7 cm, and 72.3% of patients harboring intermediate or highly complex tumors based of the RENAL nephrometry score. It is noteworthy to precise that in this > 75 yr(s) old population, age was not a predictive factor of surgical complications in our multivariate analyses. We assessed the trifecta achievement, according to Khalifeh definition, to evaluate the global surgical quality. This composite marker requires the association of negative surgical margins for cancer control, warm ischemia time under 25 min (as a surrogate of renal function preservation), and the absence of peri-operative complications^8^. We found that 45.0% of the procedures met these 3 criteria together. In their original article, Khalifeh el al compared trifecta achievement between patients receiving laparoscopic (231 patients), and robot-assisted (269 patients) PN. Patients median ages were respectively 57.7 and 58.8 yr(s) old. The Trifecta was achieved in 31.6% and 58.7% of patients respectively, with an overall 5.2% Clavien ≥ III-IV complication rate. Thus, the complication rate and overall surgical quality appear similar in our elderly patients cohort to this younger population-based study. The feasibility and safety of PN in older patients was also recently reported by Bindayi et al., who analyzed 653 over-75 yr old patients who underwent PN^14^. Using Hung’s trifecta definition^15^, they found 40.4% rate of achievement and 6.0% of Clavien ≥ III complications. Altogether, these results confirm that age should not be considered an absolute contraindication for PN. A global frailty and comorbidity assessment should be performed in older patient, and balanced with the potential risk of tumor progression on individual basis. Even after 75 yr(s) old, when a patient is fit for a surgical intervention, PN is probably the best option to control the tumor, in order to prevent CKD and quality-of-life deterioration.

The surgical approach seems to play a role in PN outcomes. In our multivariate analyses, we found that a robotic-assisted approach was the only predictive factor that prevented both medical and surgical complications. The worst renal function was associated with more surgical complications, while the tumor size and bilateral tumors were linked to more medical complications. The association between altered renal function and surgical complication could be explained with the increased risk of bleeding and developing hematoma with chronic kidney disease^16^. Larger and bilateral tumors impose longer procedure. Prolonged operative time is associated with higher risk of complications and a recent meta-analysis demonstrated a 14% increase in the likelihood of complications for every 30 min of additional operating time^17^.

This improvement with the robotic approach was already described in the overall population series. Peyronnet et al. retrospectively reviewed 1800 cases of PN that were performed in 6 academic centers, and compared robotic to the open approach. They found that the robotic approach was associated with less complications, less blood loss, and shorter hospital stay with similar oncologic outcomes^18^. Similarly, Larcher et al. reported a lower rate of overall (21% vs 36%; p < 0.0001) and major (3% versus 9%, p = 0.03) complications for those treated with robot-assisted versus open procedures. This was based on a retrospective analysis of 472 patients undergoing PN for cT1-2N0M0 renal masses. The functional and oncologic outcomes were similar in both arms^19^. Another series compared the functional and oncologic outcomes of open, laparoscopic and robot-assisted PN from 1308 patients. After an over 5-year median follow-up, the local recurrence, distant metastasis, and cancer-related death rates were similar, while CKD upstaging was lower in the robot-assisted PN arm^20^. To our knowledge, the present study is the first to demonstrate the robotic approach advantage among elderly patients in respect to post-operative complications.

The main limitations of our study are the retrospective and non-comparative design. As previously discussed, proposing a RN, when a PN is technically feasible, is hardly warrantable. It would be interesting to compare PN with active surveillance and thermal ablation in elderly patients, as to our knowledge this has never yet been performed. The aim of this study was to define the feasibility and safety of PN in an older patient population. Therefore, this endpoint did not require a comparison. Despite this, our complication rates were comparable with contemporary studies in elderly patients and in the overall PN population. Active surveillance is a valuable option for comorbid patients harboring small renal masses, and significant competing-cause of mortality^21,22^. It consists of tumor size monitoring, through regular abdominal imaging, that should trigger treatment in case of growth^4^. While large series have demonstrated usual slow tumor growth rates for patients under active surveillance and rare progressions to metastatic stages^23–25^, tumor size appears to be a reliable surrogate of tumor aggressivity^24^. Thus, AS is particularly relevant in patients with tumor sizes < 2-3 cm^26^. The same applies for ablative strategies, which can achieve acceptable oncologic outcomes for small renal masses, with good renal function preservation and low complication rates^27^. However, tumor size is the main risk factor of tumor recurrence with a reported threshold of 3-4 cm^28–31^. In our study, the median tumor size was 4.7 cm. Thus, most of the patients were probably not eligible for AS, nor ablative treatments. Despite this, our results should be confirmed by further investigations. The large number of patients included in our study, and the concordance with previous reports, confirm that age should not be a limitation when a treatment is indicated for patients harboring a RCC. Nephron-sparing surgery should be considered as the standard of care, when technically feasible, with the same quality outcomes expected as in the general population. Indications should be balanced in light of the competing comorbidities and the tumor characteristics, in order to expect benefits in terms of overall survival and quality of life.

## Conclusion

Nephron-Sparing surgery is feasible and safe, even for older patients. When they are fit for surgery, age should not be a constraint in considering this therapeutic strategy. We have demonstrated a low surgical morbidity rate in this population, comparable to the contemporary series of younger patients. It remains a valuable option, even for large tumors that are not amenable for active surveillance or ablative techniques. Faster and less morbid procedures should not be reasons to prefer RN to PN. The Robot-assisted approach seems to offer a lower rate of complications.

## Abbreviations

ASA: American society of anesthesiologists
CI: Confidence interval
CKD: Chronic kidney disease
EBL: Estimated blood loss
eGFR: Estimated glomerular filtration rate
ECOG: Eastern cooperative oncology group
LoS: Length of stay
MDRD: Modification of diet in renal disease
OR: Odds ratio
PN: Partial nephrectomy
RCC: Renal cell carcinoma
RN: Radical Nephrectomy
yr(s) old: Years old
